# In Vitro Dissolution Profile of Dapagliflozin: Development, Method Validation, and Analysis of Commercial Tablets

**DOI:** 10.1155/2017/2951529

**Published:** 2017-07-31

**Authors:** Rafaela Zielinski Cavalheiro de Meira, Aline Biggi Maciel, Fabio Seigi Murakami, Paulo Renato de Oliveira, Larissa Sakis Bernardi

**Affiliations:** ^1^Post-Graduation Program in Pharmaceutical Sciences, Department of Pharmacy, Universidade Estadual do Centro-Oeste (UNICENTRO), 85040-080 Guarapuava, PR, Brazil; ^2^Department of Pharmacy, Federal University of Paraná, Curitiba, PR, Brazil

## Abstract

Dapagliflozin was the first of its class (inhibitors of sodium-glucose cotransporter) to be approved in Europe, USA, and Brazil. As the drug was recently approved, there is the need for research on analytical methods, including dissolution studies for the quality evaluation and assurance of tablets. The dissolution methodology was developed with apparatus II (paddle) in 900 mL of medium (simulated gastric fluid, pH 1.2), temperature set at 37 ± 0.5°C, and stirring speed of 50 rpm. For the quantification, a spectrophotometric (λ = 224 nm) method was developed and validated. In validation studies, the method proved to be specific and linear in the range from 0.5 to 15 *μ*g·mL^−1^ (*r*^2^ = 0.998). The precision showed results with RSD values lower than 2%. The recovery of 80.72, 98.47, and 119.41% proved the accuracy of the method. Through a systematic approach by applying Factorial 2^3^, the robustness of the method was confirmed (*p* > 0.05). The studies of commercial tablets containing 5 or 10 mg demonstrated that they could be considered similar through *f*1, *f*2, and dissolution efficiency analyses. Also, the developed method can be used for the quality evaluation of dapagliflozin tablets and can be considered as a scientific basis for future official pharmacopoeial methods.

## 1. Introduction

**Figure 1:**
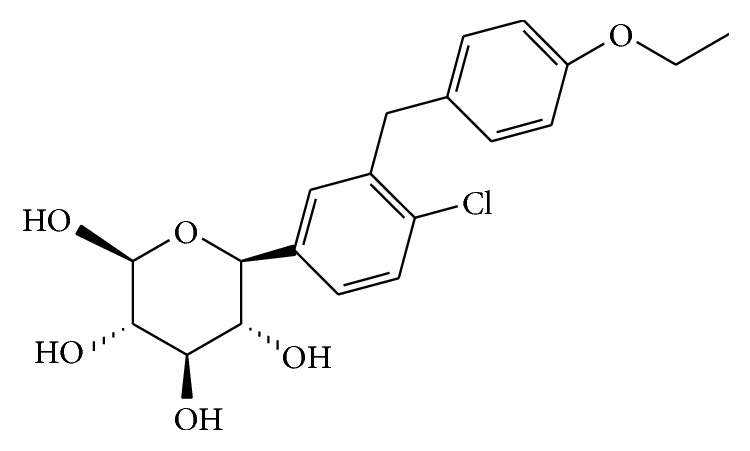
Chemical structure of dapagliflozin (DAPA).

The dapagliflozin (DAPA) is a highly potent, selective, and reversible inhibitor of sodium-glucose cotransporter 2 (SGLT2). It acts by reducing the reabsorption of glucose by the kidney, leading to excretion of excess glucose in the urine, thereby improving glycemic control in patients with type 2 diabetes mellitus [1]. DAPA (Figure 1) is chemically described as (1S)-1,5-anhydro-1-C-[4-chloro-3-[(4-ethoxyphenyl) methyl]phenyl]-D-glucito. It is a white crystalline powder, soluble in ethanol, methanol, dimethylsulfoxide (DMSO), and dimethylformamide [2]. According to the European Medicines Agency (EMA), DAPA is classified as Class III in the Biopharmaceutics Classification System (BCS), being highly soluble and poorly permeable [3].

This drug was the first of its class to be approved by the European Union [4] and the commercial formula, Forxiga®, was approved by the US Food and Drug Administration (FDA) in January 2014. In Brazil, the approval of the Health Surveillance Agency (ANVISA) occurred in July 2013 [5, 6].

The dissolution study is extremely important in assessing the performance of a solid pharmaceutical formulation and considering that the literature does not report any study regarding the dissolution profile of DAPA and that there is no pharmacopoeial method approved for dissolution evaluation, the aim of this work was to develop and validate a dissolution methodology to ensure the quality of the tablets in the market and serve as a guidance for a future pharmacopoeial monography. The quantification was carried out by UV-Vis spectrophotometry due to its high throughput capacity and low cost that were considered as great advantages for employment in routine quality control laboratories.

## 2. Material and Methods

The DAPA propanediol monohydrate raw material was obtained from Lianchuang Biological Pharmaceutical Co. (Anhui, China, Batch: 20150207). Five lots of Forxiga (AstraZeneca, Cotia, SP, Brazil) tablets, two lots containing 5 mg of DAPA (identified in this study as lots A and B) and three lots containing 10 mg of DAPA (C, D, and E), were bought in commercial pharmacies.

### 2.1. Dissolution Studies

For the preparation of the reference stock solution of DAPA, 10 mg was weighted out and diluted to volume with methanol into a 10 mL volumetric flask. The solution was stored in refrigerator (4°C) and protected from light.

The dissolution method was developed in a dissolution equipment (708 DS, Agilent) with apparatus II (paddle) in 900 mL of medium and fixed temperature of 37 ± 0.5°C. The tested media were as follows: simulated gastric fluid without enzymes pH 1.2, simulated intestinal fluid without enzymes pH 6.8, acetate buffer pH 4.5, ultrapure water (Milli-Q®, millipore), and HCl 0.1 M. All dissolution media were previously degassed by vacuum filtration. Agitation speeds of 50, 60, and 75 rpm were also evaluated [7]. For the quantification, a UV-Vis spectrophotometer was used at 224 nm (Carry 100, Agilent). The study of the influence of filters was carried out by evaluating quantitative paper 28 *μ*m (J. Prolab), nylon syringe filter 0.22 *μ*m (Sterlitech), and the Full Flow® filter 10 *μ*m (Agilent).

### 2.2. Validation of Analytical Methodology

The analytical dissolution methodology was validated by analyzing the parameters required by ICH Q2 (R1) and by USP 38, NF 33, Chapter 1092 (the dissolution procedure: development and validation), through specificity, linearity, limit of detection (LOD), limit of quantitation (LOQ), accuracy, precision, and robustness [8, 9]. In order to confirm the applicability of the methodology, the study of the dissolution profile of different commercial batches of DAPA was carried out.

#### 2.2.1. Specificity

The specificity was carried out in order to demonstrate that there was no interference from placebo constituents in the analysis [9]. The DAPA standard solution dissolved in methanol was added to a vessel containing the dissolution medium, to obtain a theoretical final concentration of 5.556 *μ*g·mL^−1^. The powdered excipients were also added in vessels containing the medium. The experiments were carried out in triplicate and the rotation speed was set to 150 rpm. Aliquots of these samples were taken after 30 minutes of dissolution and the absorbance analyzed by UV-Vis spectrophotometry.

#### 2.2.2. Linearity

Linearity was prepared from three standard solutions. Initially, an amount of 10 mg of DAPA was exactly weighted out and diluted in 10 mL volumetric flask with methanol, to obtain a final concentration of 1 mg·mL^−1^. This solution was then diluted in six levels (0.5, 1, 2, 5, 10, and 15 *μ*g·mL^−1^) with dissolution media covering the lowest and the highest concentration that were expected in the release of the drug during dissolution. This parameter was calculated using linear regression [9].

#### 2.2.3. Limits of Detection and Quantitation

The limits of detection and quantification were calculated by the curve slope and standard deviation obtained from the average of the intercepts of the curves made in triplicate in the evaluation of linearity [8]. The values obtained were confirmed experimentally.

#### 2.2.4. Accuracy

The accuracy was evaluated by the addition of the standard solution and excipients to the same vessel, to obtain the final concentrations of 4.445, 5.556, and 6.667 *μ*g·mL^−1^ corresponding to 80, 100, and 120% of the concentration defined for the validation, respectively. The absorbance values obtained that should present recovery were 95 to 105% relative to a standard solution at the concentration defined for validation [9].

#### 2.2.5. Precision

The precision was determined by calculating the standard deviation from the dissolution of six tablets. The two dosages of DAPA tablets (5 and 10 mg) were used for precision evaluation. The analyses were performed on different days (interday) and by different analysts (between-analysts). The acceptance criterion was that the RSD values should be lower than 2% [9].

#### 2.2.6. Robustness

The robustness was studied by analyzing small changes in the dissolution conditions, such as pH (1.20 ± 0.2), salt (NaCl) concentration (2.00 ± 0.10 g), and volume of the dissolution medium (900 ± 50 mL). The rotation speed was kept at 50 rpm and sampling time was 30 min. By following the recommendations of the new FDA Guidance for Analytical Procedures and Methods for Validation of Drugs and Biological Products, we applied a systematic approach using a Factorial 2^3^ model (Statistica® V.8 software) for the selection of design points [10]. The statistical analyses were performed using ANOVA and, to be valid, the changes should not be significant (*p* > 0.05).

### 2.3. Dissolution Profile Study

The dissolution profile study was performed with two batches of 5 mg DAPA (identified as A and B) and three lots of 10 mg DAPA (identified as C, D, and E). For each batch, twelve tablets were used. The dissolution sampling times were 0.5, 1, 1.5, 2, 3, 4, 5, 10, and 20 min. For each time point, 10 mL of sample was withdrawn and immediately replaced with fresh medium. The samples were immediately filtered through a quantitative paper filter and quantitated by UV-Vis spectroscopy.

The dissolution profile was also analyzed by applying factors *f*1 (difference factor (1)) and *f*2 (similarity factor (2)), setting randomly one of the batches of each dosage (5 mg: A; 10 mg: D) as the reference [11]. These factors, proposed by Moore and Flanner (1996) [12], are specific models for the comparison between the profiles.

(1) f1=∑t=1nRt−Tt∑t=1nRt×100,

(2) f2=50log⁡1+1n∑t−1nRt−Tt2−0.5×100.

The dissolution profiles can be considered similar if the values of *f*1 are between 0 and 15, and if *f*2 found are in the range of 50 to 100 [12, 13].

The dissolution efficiency (DE) was also applied in the comparative analysis of the profiles. This model statistically evaluates the equivalence of the dissolution tests across the area under the curve obtained from the percent dissolution of the drug over the time [14]. The results were analyzed using Student's *t*-test for lots of DAPA 5 mg and ANOVA for lots of DAPA 10 mg, with significance level of 0.05%.

## 3. Results and Discussion

### 3.1. Dissolution Studies

The development of the dissolution method was carried out with the media previously degassed by vacuum filtration (0.44 *μ*m cellulose membrane). If the air dissolved in the dissolution medium is not properly eliminated, air bubbles can act as a barrier to the dissolution process (if present on the tablet surface) and can adversely affect the reliability of the results. Furthermore, bubbles can cause particles to cling to the apparatus and vessel walls. Bubbles on the dosage unit may increase buoyancy, leading to an increase in the dissolution rate, or may decrease the available surface area, leading to a decrease in the dissolution rate.

The apparatus used in the tests was the paddle (apparatus II). Its use is recommended when the tests are performed with immediate release tablets [9]. The filter test did not show any significant interference, since the assay of the samples filtered in quantitative paper filter (28 *μ*m), Full Flow filter (10 *μ*m), and nylon syringe filter (0.22 *μ*m) was 100.38, 99.23, and 98.85%, respectively. Since all variations were within ±2%, the quantitative paper filter paper was chosen for economical reason.

**Figure 2:**
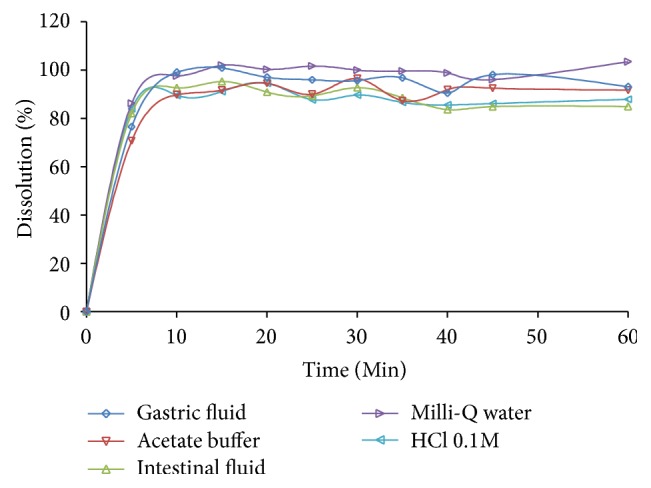
Dissolution profile of DAPA in different dissolution media at 50 rpm.

Among the analyzed media, the simulated gastric fluid was selected for the validation process. In less than 10 minutes in this medium, the drug was practically totally dissolved, presenting increasing dissolution percentage from 5 to 15 minutes and thus remaining constant until the last sampling time, as shown in Figure 2. Additionally, these media are more predictive of the in vivo behavior of the drug after oral administration. The Milli-Q water also showed excellent results, but it is not among the media indicated by the United States Pharmacopeia [9] since the quality of the water may vary from laboratory to laboratory, thus impairing the reproducibility of results.

The results obtained during the development of the analytical methodology for dissolution of DAPA tablets showed that this drug presented a high dissolution percentage (>85% of the drug dissolved in 15 minutes of test, at 50 rpm in simulated gastric fluid). These results corroborate the BCS data found in the literature that classifies DAPA as a Class III drug [3].

The lower rotation speed (50 rpm) was chosen for presenting more discriminative results at the beginning of the dissolution, assisting in the observation of the profile. The recommendation from the USP using apparatus II, for the dissolution of immediate release tablets, is that the agitation speed should be between 50 and 75 rpm [9]. The other stirring velocities also showed high dissolution percentage results with the simulated gastric fluid medium, which was verified by statistical analysis of the data (*f*1 and *f*2), comparing the lowest rotation with the other velocities studied. The difference factor (*f*1) was 9.80 for 60 rpm and of 9.09 for 75 rpm, and the similarity factor (*f*2) was of 52.75 for 60 rpm and 56.94 for 75 rpm, always compared to 50 rpm. However, with a slower rotation speed, there is also the advantage of more easily detecting any deviation of quality in industrial production.

### 3.2. Validation of Analytical Methodology

In validation, the method proved to be specific and the possible interference from excipients was of 1.81%, not exceeding the limit of 2%, at the selected wavelength, compared to a standard solution of DAPA.

The linearity was analyzed in the range from 0.5 to 15 *μ*g·mL^−1^ (*y* = 0.052*x* − 0.008, *r*^2^ = 0.998). The analysis of variance (ANOVA) was performed, demonstrating a significant linear regression (*p* < 0.05) and no significant deviation from linearity (*p* > 0.05), validating the assay. The limits of detection and quantification calculated and confirmed experimentally were 0.05 and 0.15 *μ*g·mL^−1^, respectively.

Considering the precision-repeatability analysis, the dissolution of six tablets (30 min.) under the same conditions resulted in the RSD of 1.39 and 0.67% for the 5 and 10 mg tablets, respectively (Table 1). The interday precision obtained for the 5 mg dosage was 0.11% (RSD) and for the 10 mg dosage was 0.41% (RSD). The between-analysts (two analysts) analysis resulted in RSD of 0.15% for the 5 mg tablets and 1.09% for 10 mg DAPA tablets. Since all RSD values were lower than 5%, the method was considered precise for both dosages.

The accuracy showed a recovery of 4.485, 5.470, and 6.634 *μ*g·mL^−1^ of the DAPA standard solution added in the vessel containing all excipients, corresponding to 80.72, 98.47, and 119.41%, respectively, with RSD of 0.73, 0.67, and 1.03% for each level. The results together with the absorbance values are shown in Table 2.

**Figure 3:**
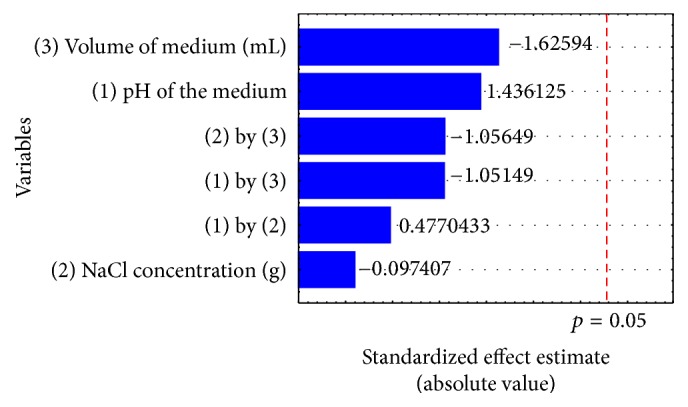
Variables implemented in the study of the robustness versus the absolute value of the effects.

Through a systematic approach by applying Factorial 2^3^, the robustness of the method was confirmed (Table 3, Figure 3). The Factorial model was validated (*F*_calc_ = 2.11 > *F*_tab_ = 0.55) and, in the analysis of the results (ANOVA), no significant values (*p* > 0.05) were found. This type of tool has been widely used in different areas allowing the selection of experimental points to prove that small variations do not influence the results or even to find the best point for a given response [15]. Although robustness is not mandatory for validation procedure, its inclusion in the protocol is useful for understanding the range within the method is suitable.

### 3.3. Method Application

**Figure 4:**
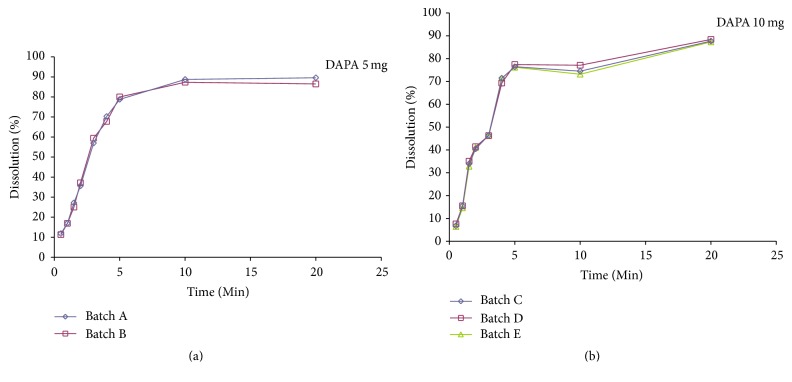
Dissolution profile of two batches of Forxiga 5 mg (a) and three bathes of Forxiga 10 mg (b).

Five commercial tablets containing DAPA were analyzed and the results are shown in Figure 4. Although visually the dissolution profiles of Forxiga 5 mg were different from the Forxiga 10 mg, when we consider the same dosage (5 or 10 mg), the profiles were very similar.

For the 5 mg tablets, lot A was compared to lot B, resulting in *f*1 lower than 15 (1.92) and *f*2 higher than 50 (87.52), determining similarity of behavior between them. Regarding the 10 mg tablets, lot D when compared to C showed *f*1 lower than 15 (1.15) and *f*2 higher than 50 and very close to 100 (93.41), and when compared to lot E, it also presented *f*1 lower than 15 (1.41) and *f*2 higher than 50 (91.63), confirming that the batches exhibited the same dissolution behavior in vitro.

The dissolution efficiency study for the comparative analysis of the dissolution profiles also revealed similarity between them. The results were (mean ± standard deviation) as follows: 76.49 ± 0.66 for lot A, 75.78 ± 0.47 for lot B, 70.38 ± 0.31 for lot C, 70.92 ± 0.68 for lot D, and 70.25 ± 0.84 for lot E. The *t*-test used in the data treatment for the DAPA 5 mg lots and the ANOVA for the 10 mg lots did not show significant differences between the means of dissolution percentages obtained (*p* > 0.05).

## 4. Conclusion

The dissolution and the quantification methodologies were developed and validated according to the requirements of ICH and USP. The following dissolution conditions were considered optimized: apparatus II (paddle), 900 mL of medium (simulated gastric fluid, pH 1.2), temperature of 37 ± 0.5°C, and stirring speed of 50 rpm. The analysis of different commercial batches containing 5 or 10 mg of dapagliflozin demonstrated a similar dissolution profile (considering the same dosage). The developed methods could be considered as a scientific basis for future official pharmacopoeial methods, for the routine quality control in the pharmaceutical industry and for studies where the dapagliflozin dissolution is required.

## Figures and Tables

**Table 1:** Repeatability of the analytical method for the in vitro dissolution of dapagliflozin.

	Repeatability (*n* = 6)			
	Tablets 5 mg		Tablets 10 mg	
	Absorbance	Assay (%)	Absorbance	Assay (%)
	0.266	101.92	0.439	100.46
	0.264	101.15	0.441	100.92
	0.273	104.60	0.442	101.14
	0.264	101.15	0.441	100.92
	0.267	102.30	0.447	102.29
	0.271	103.83	0.445	101.83

RSD (%)	1.39		0.67	

**Table 2:** Accuracy of the analytical method for the in vitro dissolution of dapagliflozin.

Analysis	Absorbance (80%)	Absorbance (100%)	Absorbance (120%)
1°	0.212	0.259	0.314
2°	0.209	0.256	0.308
3°	0.211	0.256	0.313

RSD (%)	0.73	0.67	1.03

Recovery (%)	80.72	98.47	119.41

**Table 3:** Robustness test (Factorial analysis 2^3^) of the analytical method for the in vitro dissolution of dapagliflozin.

pH of the medium	NaCl concentration (g)	Volume of medium (mL)	Drug released after 30 mim (%)
1.00	2.10	850	100.00
1.40	1.90	850	101.53
1.40	1.90	950	98.47
1.00	1.90	950	100.00
1.40	2.10	850	101.53
1.40	2.10	950	99.23
1.00	2.10	950	96.93
1.00	1.90	850	98.08
1.20	2.00	900	101.15
1.20	2.00	900	99.23
1.20	2.00	900	100.77
